# Predicting Overweight and Obesity Status Among Malaysian Working Adults With Machine Learning or Logistic Regression: Retrospective Comparison Study

**DOI:** 10.2196/40404

**Published:** 2022-12-07

**Authors:** Jyh Eiin Wong, Miwa Yamaguchi, Nobuo Nishi, Michihiro Araki, Lei Hum Wee

**Affiliations:** 1 Centre for Community Health Studies Faculty of Health Sciences Universiti Kebangsaan Malaysia Kuala Lumpur Malaysia; 2 National Institute of Health and Nutrition National Institutes of Biomedical Innovation, Health and Nutrition Tokyo Japan; 3 Faculty of Health and Medical Sciences School of Medicine Taylor's University Selangor Malaysia

**Keywords:** overweight, obesity, prediction, machine learning, logistic regression, etiology, algorithms, Malaysia, adults, predictive models, accuracy, working adults, surveillance

## Abstract

**Background:**

Overweight or obesity is a primary health concern that leads to a significant burden of noncommunicable disease and threatens national productivity and economic growth. Given the complexity of the etiology of overweight or obesity, machine learning (ML) algorithms offer a promising alternative approach in disentangling interdependent factors for predicting overweight or obesity status.

**Objective:**

This study examined the performance of 3 ML algorithms in comparison with logistic regression (LR) to predict overweight or obesity status among working adults in Malaysia.

**Methods:**

Using data from 16,860 participants (mean age 34.2, SD 9.0 years; n=6904, 41% male; n=7048, 41.8% with overweight or obesity) in the Malaysia’s Healthiest Workplace by AIA Vitality 2019 survey, predictor variables, including sociodemographic characteristics, job characteristics, health and weight perceptions, and lifestyle-related factors, were modeled using the extreme gradient boosting (XGBoost), random forest (RF), and support vector machine (SVM) algorithms, as well as LR, to predict overweight or obesity status based on a BMI cutoff of 25 kg/m^2^.

**Results:**

The area under the receiver operating characteristic curve was 0.81 (95% CI 0.79-0.82), 0.80 (95% CI 0.79-0.81), 0.80 (95% CI 0.78-0.81), and 0.78 (95% CI 0.77-0.80) for the XGBoost, RF, SVM, and LR models, respectively. Weight satisfaction was the top predictor, and ethnicity, age, and gender were also consistent predictor variables of overweight or obesity status in all models.

**Conclusions:**

Based on multi-domain online workplace survey data, this study produced predictive models that identified overweight or obesity status with moderate to high accuracy. The performance of both ML-based and logistic regression models were comparable when predicting obesity among working adults in Malaysia.

## Introduction

Overweight and obesity are global health issues that are increasingly recognized as major public health concerns in low- and middle-income countries. In Malaysia, 1 in 2 adults, particularly those of working age (ie, aged 30 to 65 years), is either overweight or obese [1]. This is concerning, as obesity prevalence is rising at a very high rate (3.3%) in this country [2]. The increase in overweight and obesity is related to increases in noncommunicable diseases, the mortality rate, and health care costs, as well as decreases in productivity and economic growth [2-5].

Obesity is a chronic, relapsing, multifactorial disease that is attributable to individual or biological, psychological, sociocultural, local, and global environmental factors [6-8]. As obesity is largely preventable, understanding the determinants of and risk factors for obesity is important for the development of population-based strategies to prevent obesity. Identifying individuals at high risk of obesity enables early intervention to modify obesity risk factors. Conventional statistical methods, such as generalized linear or regression models with a low number of predictor variables, have been successful in identifying obesity [9]. However, given the complexity of the etiology of obesity, regression modeling may not be adept at disentangling nonlinear and interdependent relationships among factors for obesity prediction.

Machine learning (ML) is an advanced data analytical method that uses fine-tuned algorithms to characterize and predict outcomes by learning from data without being explicitly programmed to do so. As health data become more available and accessible, ML techniques are increasingly used to perform such complex tasks in obesity research as classifying and predicting obesity at individual and group levels [10-12]. ML techniques have advantages over regression modeling, as they are data driven and do not necessitate a priori assumptions, such as normality, linearity, and multicollinearity. In addition, ML techniques are capable of handling high-dimensional and complex data sources beyond numeric sources, and therefore may be able to provide new insights into unexplored predictor variables [9,13]. Thus, ML techniques are likely to be more accurate than regression models in obesity prediction [14].

A wide range of ML-based algorithms incorporating various predictors and risk factors, training set sizes, and degrees of implementation have been used to predict adult obesity [11,14]. The reported accuracy of ML algorithms to predict adult obesity as a binary outcome ranges broadly, from 0.59 to 0.97 for overall accuracy [15-24] and 0.51 to 0.99 for the area under the curve (AUC) [15,19,20,23,24]. A review suggested that ML-based models predicted childhood and adolescent obesity much better than linear regression [13]. However, studies that have compared the performance of different ML algorithms with regression in adult obesity have reported mixed findings. Some evidence suggests superior performance for ML models compared to regression models [19,21], while some suggests similar or inferior performance [15,17,18,23]. These inconsistencies may partly be due to data quality, variable selection, and the use of different approaches to model fitting, parameter tuning, and validation among studies.

The Malaysia’s Healthiest Workplace by AIA Vitality survey is a large, observational online survey of the health and well-being of Malaysian employees [25]. Since 2017 (with the exceptions of 2020 and 2021, because of the COVID-19 pandemic), this annual online workplace survey has collected comprehensive information on Malaysian employees’ sociodemographic characteristics, physical and mental health, smoking and alcohol habits, physical activity, diet, musculoskeletal health, and work environment as a database to inform workplace interventions and improve productivity [25]. In this study, we propose an ML-based model to predict overweight and obesity status among employees in Malaysia based on multi-domain variables collected in this large survey. We evaluated the performance of 3 ML algorithms and compared them with logistic regression for the prediction of overweight and obesity status. We hypothesized that ML algorithms would outperform logistic regression models in predicting overweight and obesity status based on BMI.

## Methods

### Study Design and Data

This is a retrospective study of predictive model derivation using data from the Malaysia’s Healthiest Workplace by AIA Vitality 2019 survey. This online survey, commissioned by AIA Malaysia and delivered in partnership with RAND Europe, was administered between May and August 2019. The survey, which has taken place annually in Malaysia from 2017 to 2019, aimed to determine workplace productivity and multi-domain factors that influence workplace productivity. Employees from small, medium, and large organizations were invited to answer a 40-minute employee survey questionnaire about their general health, lifestyle behaviors, mental health status, and work environment. The study rationale and methodology have been discussed in detail elsewhere [26-28].

**Figure 1:**
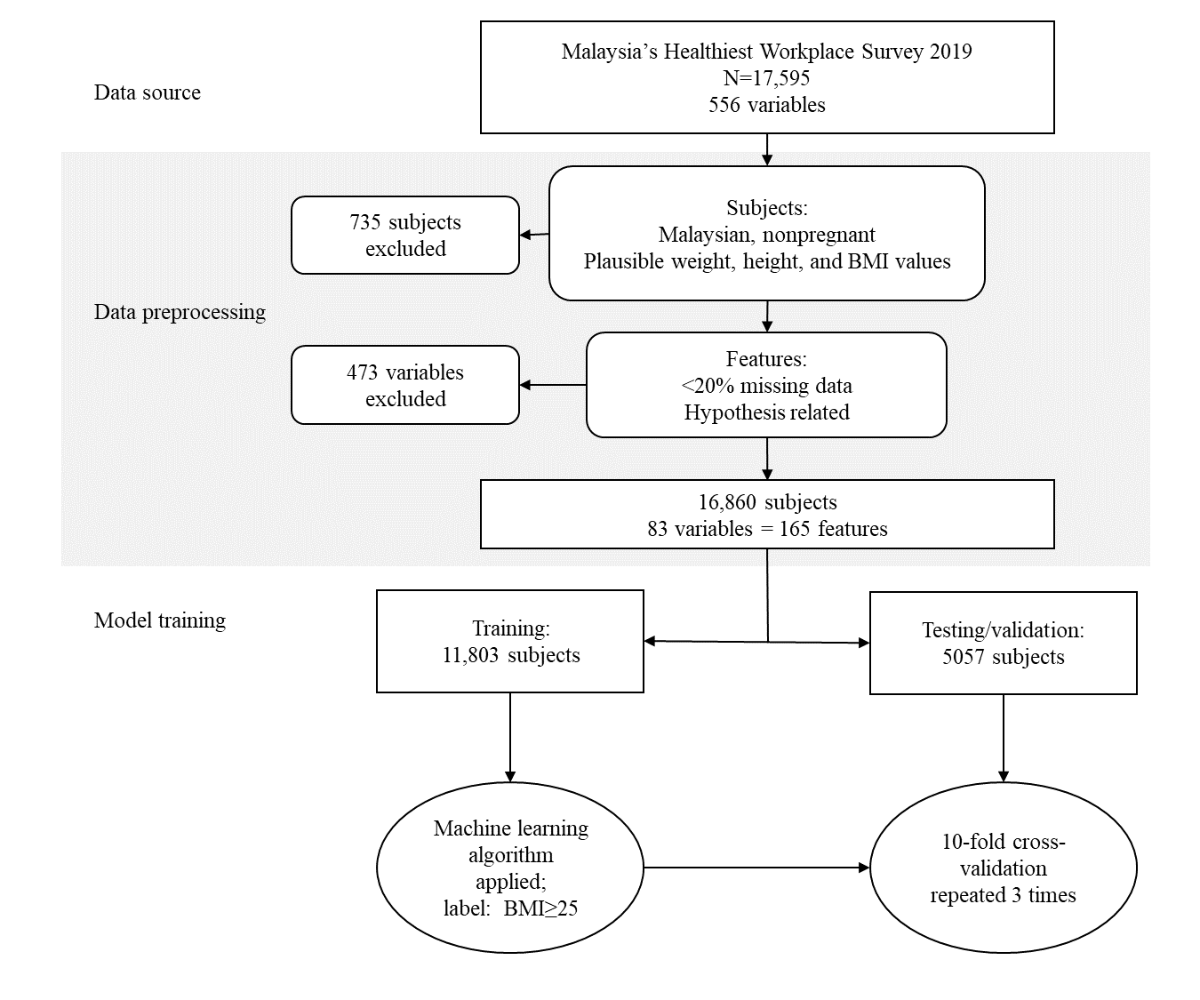
Overview of data preprocessing, model development, and model evaluation.

The initial data set comprised data submitted by 17,595 participants from 230 companies. We initially included 16,931 participants resident in Malaysia for whom data were available for body weight and height. If they were women, participants were included if they were not pregnant. Participants with (1) body weight more than 200 kg, (2) height more than 200 cm, or (3) BMI values of more than 60 kg/m^2^ or less than 14 kg/m^2^ were deemed to have implausible values and were excluded from analysis. After excluding 71 participants who reported implausible weight, height, or BMI, the final data set included 16,860 of 16,931 participants (95.8%) (Figure 1).

### Ethics Approval

The use of the data was approved by the Research Ethics Committee Universiti Kebangsaan Malaysia (JEP-2020-707). As the obtained pooled data were anonymized and deidentified, informed consent from the participants was not required. The study results were presented following the reporting guidelines and recommendations for ML [29,30].

### Data Preprocessing

An overview of data preprocessing and model development is illustrated in Figure 1. Data preprocessing involved the selection of participants and variables (features) followed by mean substitution of missing data, one-hot encoding of categorical variables, and min-max scaling for data normalization.

### Outcome Variable

The outcome of interest was overweight or obesity status, defined as a BMI of 25 kg/m^2^ or more [31]. This was calculated by dividing the self-reported body weight (in kg) by the squared height (in m^2^). The cutoff of 25 was chosen as Southeast Asians are reported to have higher body fatness at a lower BMI than Europeans [32,33] and are therefore predisposed to elevated cardiovascular risk factors and other adverse effects of obesity at lower BMI ranges (23 kg/m^2^ to 25 kg/m^2^), as observed in local studies [34,35]. Further, a recent study suggested that a BMI of 24.8 kg/m^2^ is an optimal BMI cutoff to define obesity among Malaysian adults based on percentage of body fat [36].

### Predictor Variables

Initially, the data set consisted of 556 predictor variables. A total of 473 variables that contained redundant information or text information with more than 20% missing or nonapplicable data were removed from the data set. The reduced data set included 83 variables that were grouped into the following 4 main domains: sociodemographic characteristics, job characteristics, status perception, and lifestyle-related behaviors (the list of predictor variables is included in Multimedia Appendix 1).

Categorical variables (n=16) were one-hot encoded into binary variables. For instance, weight satisfaction was assessed by a categorical question that prompted participants to select 1 of 3 statements that best described how they felt about their current body weight. The participants indicated whether they (1) were happy with their weight, (2) were not happy with their weight but had no intention of losing or gaining weight, or (3) wanted to change their weight. This categorical variable was subsequently encoded into 3 binary variables (ie, “weight_satisfaction_1,” “weight_satisfaction_2,” and “weight_satisfaction_3”). Finally, prediction models were trained and tested on the final 165 normalized variables. A total of 120 (73%) of these 165 predictor variables were binary (yes/no) variables.

### Statistical Analysis Methods

#### Model Development

The R (version 3.6.1; R Software Foundation) package “caret” (version 6.0-90) was used for model training and validation [37]. Based on a random 70:30 split, a total of 11,803 participants, including 4934 (41.8%) with overweight or obesity, were used to train the model. The remaining 30% of the participants (5057/16,860) ware used to predict the obesity outcome during model validation.

Three supervised, nonlinear ML classifiers were applied, namely extreme gradient boosting (XGBoost), random forest (RF), and a support vector machine (SVM). XGBoost is a tree-based ensemble algorithm that uses a boosting method to create multiple decision trees sequentially. The algorithm combines the predictions of weak decision trees to produce a more robust final model. Improvised on the gradient boosting framework, XGBoost is a popular learning algorithm due to its high predictive power and efficiency in handling continuous and categorical data using relatively low computational power [38]. RF is also an ensemble method but uses a bagging method to train multiple decision trees in parallel using random selection of predictors. The final model merges predictions from each decision tree to predict a class [39]. Finally, SVMs use a kernel-based algorithm to construct a decision boundary or hyperplane that best separates the data into 2 classes in n-dimensional space. SVMs use extreme cases, also known as support vectors, to create an optimal hyperplane that has the maximum margin between the vectors [40].

In this study, logistic regression (LR) was compared with the 3 ML models. Logistic regression is a part of the generalized linear model and is the conventional classifier for categorical outcome responses. The algorithm assumes a linear relationship between the predictor variables and the log odds (probability) of obesity as the outcome in this study. All predictor variables were included in the model, regardless of statistical significance, to maintain comparability across models. The goodness of fit of the logistic regression model was demonstrated by a McFadden *R*^2^ value of 0.3452 and a Nagelkerke *R*^2^ value of 0.3452. The probability produced by the logistic regression was subsequently assigned to a binary outcome (overweight/obese or not), based on the customary probability cutoff point of 0.5.

The details of the package, functions, and parameters used in this study are presented in Multimedia Appendix 2. Using a grid search approach, the best combinations of parameters were employed for each algorithm. All models were tuned using 10-fold cross-validation repeated 3 times. Using the varImp function of the caret library, model-specific metrics were used to identify the best-performing predictors. To present the relative ranking of each predictor, the measures of importance for all models were scaled to have a maximum value of 100.

#### Model Evaluation

The final trained models were saved and restored for prediction using a separate test data set (n=5057) and for comparison with other models. Classification metrics were obtained from the confusion matrix (confusionMatrix) embedded in the caret package. A prediction of overweight or obesity status was considered a positive prediction. Performance was assessed by 4 main metrics (the first 3 metrics are limited in their discriminating power in selecting the best classifier [41], but they are the most common metrics used in the literature and are therefore presented for comparison with other studies): (1) accuracy, the proportion of correct predictions divided by the total number of instances evaluated; (2) sensitivity (also known as the true positive rate), the proportion of actual positives (ie, overweight or obese status) that were correctly predicted; (3) specificity (also known as the true negative rate), the proportion of actual negatives (ie, no overweight or obese status) that were correctly predicted; and (4) AUC, which represents a tradeoff between sensitivity and specificity and served as the main metric for model evaluation. AUC is extracted from the receiver operating characteristic (ROC) curve, which is the probability plot of the true positive rate (ie, sensitivity) against the false positive rate (ie, 1–specificity). An AUC above 0.5 indicates the model is better capable of distinguishing positives (ie, subjects with overweight or obesity) from negatives. In general, an AUC of 0.7 to <0.8 is considered acceptable, 0.8 to <0.9 excellent, and 0.9 or above outstanding predictive performance [42]. The ROCs and corresponding AUCs were computed and plotted with the pROC package.

The performance metrics of all predictive models are presented as point estimates with 95% CIs. For accuracy, sensitivity, and specificity, 95% CIs were calculated assuming a Gaussian distribution of the proportion. For AUCs, 95% CIs were derived through resampling with the bootstrap percentile method with 2000 repetitions. Model comparisons were made based on the 95% CIs of the 4 performance metrics.

## Results

### Study Characteristics

The analysis included 16,860 participants, of whom 41% (n=6904) were male and 41.8% (n=7048) had overweight or obese status. The male participants were significantly older, and the distributions for ethnicity, education level, marital status, occupation, individual monthly income, and obesity status were also significantly different by sex (*P*<.001 for all; Multimedia Appendix 3).

### Model Comparisons

**Figure 2:**
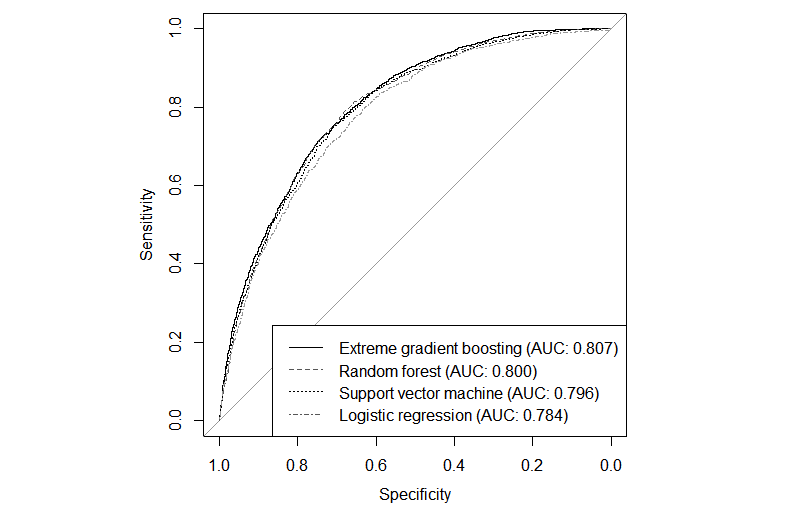
Receiver operating characteristic curves with corresponding AUC values; AUC values for each model are also presented in Table 2. AUC: area under the curve.

Table 1 presents the predictive performance of the ML and logistic regression models. Among the 4 models, the RF and LR models had lower sensitivity but higher specificity. While XGBoost exhibited the best mean accuracy and AUC, overall accuracy was similar across all models based on the 95% CIs. The ROCs of the 4 models are illustrated in Figure 2.

Table 2 compares the performance of XGBoost and LR in predicting obesity by sex. For both algorithms, the models for female participants recorded higher specificity but lower sensitivity than the models for male participants. Overall accuracy and AUC were similar across all 4 models, with the 2 algorithms showing no sex-specific differences in predictive performance.

**Figure 3:**
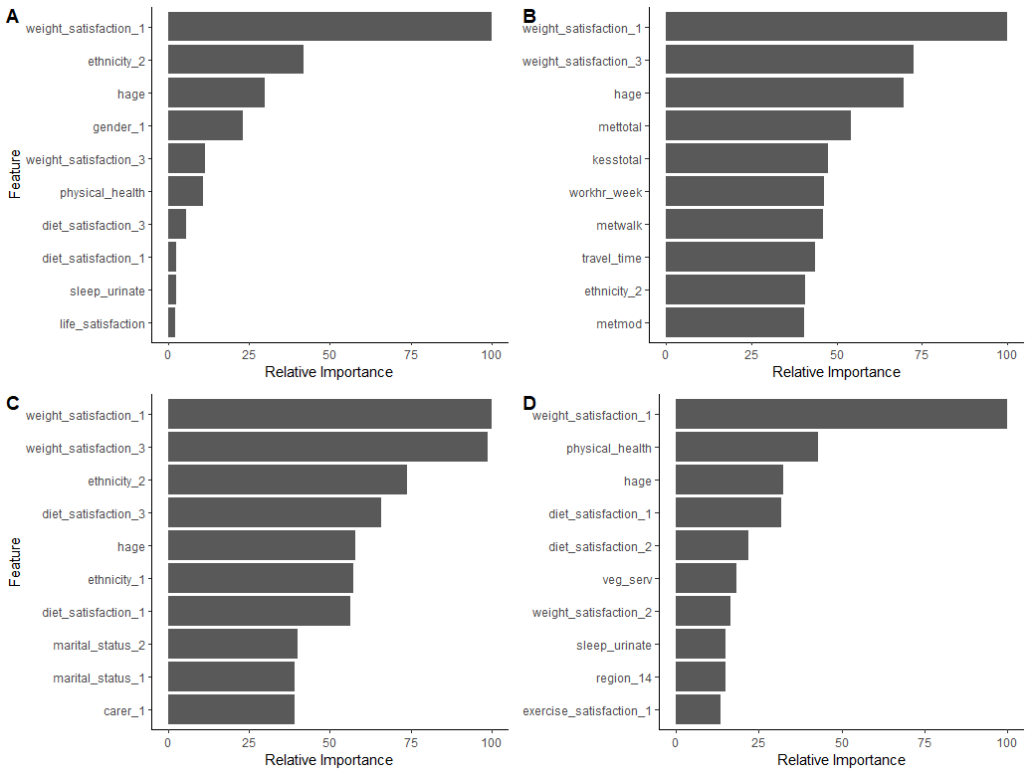
Variable importance plots of obesity predictors for extreme gradient boosting (A), random forest (B), support vector machine (C) and logistic regression (D) models. The top 10 predictors are shown for all models.

The ranking of the most important predictors of the models is summarized in Figure 3. In order of importance, the top 4 predictor variables for the XGBoost ML model were weight satisfaction, ethnicity, age, and gender. For the LR model, the top predictor variables were weight satisfaction, physical health, age, and diet satisfaction.

**Table 1:** Performance of machine-learning algorithms and logistic regression in obesity prediction.

Metrics	Gradient boosting, mean (95% CI)	Random forest, mean (95% CI)	Support vector machine, mean (95% CI)	Logistic regression, mean (95% CI)
Accuracy^a^	0.73 (0.72-0.75)	0.73 (0.71-0.74)	0.72 (0.71-0.73)	0.71 (0.70-0.72)
Sensitivity^a^	0.67 (0.65-0.69)	0.60 (0.58-0.62)	0.65 (0.62-0.67)	0.56 (0.54-0.58)
Specificity^a^	0.78 (0.76-0.79)	0.82 (0.80-0.83)	0.77 (0.76-0.79)	0.82 (0.81-0.83)
Area under the curve^b^	0.81 (0.79-0.82)	0.80 (0.79-0.81)	0.80 (0.78-0.81)	0.78 (0.77-0.80)

^a^In these rows, 95% CIs were calculated assuming Gaussian distribution of the proportions.

^b^In this row, 95% CIs were derived through resampling with the bootstrap percentile method with 2000 repetitions.

**Table 2:** Comparison of performance between machine learning and logistic regression in sex-specific obesity prediction.

	Gradient boosting, mean (95% CI)		Logistic regression, mean (95% CI)	
Metrics	Male participants	Female participants	Male participants	Female participants
Accuracy^a^	0.71 (0.69-0.73)	0.74 (0.72-0.75)	0.70 (0.68-0.72)	0.73 (0.71-0.74)
Sensitivity^a^	0.75 (0.73-0.78)	0.61 (0.58-0.63)	0.72 (0.69-0.75)	0.60 (0.57-0.63)
Specificity^a^	0.66 (0.63-0.69)	0.81 (0.80-0.83)	0.68 (0.65-0.71)	0.80 (0.78-0.81)
Area under the curve^b^	0.78 (0.76-0.80)	0.81 (0.79-0.82)	0.76 (0.74-0.78)	0.79 (0.77-0.80)

^a^In these rows, 95% CIs were calculated assuming Gaussian distribution of the proportions.

^b^In this row, 95% CIs were derived through resampling with the bootstrap percentile method with 2000 repetitions.

## Discussion

### Principal Results

This study applied various ML models and compared their performance to the performance of a conventional logistic regression model in predicting overweight or obesity status among working adults in Malaysia. Our results showed that ML and logistic regression had similarly acceptable or excellent predictive performance, as assessed by the metrics of accuracy (values ranged from 70% to 75%) and AUC (values ranged from 78% to 81%), for both the overall and sex-specific models.

### Comparison With Prior Work

Our findings, based on data collected annually as part of a large-scale online survey of employees, compare favorably to those of a recent study by Thamrin et al [23] that also used a large Southeast Asian sample (N=618,898), in Indonesia. That study employed logistic regression, classification and regression trees, and a naive Bayes classifier for obesity prediction based on data for sociodemographic characteristics, diet, physical activity, lifestyle behaviors, and health status from the Indonesian Basic Health Research periodic survey. The study reported accuracy between 70.8% and 72.2% and an AUC between 0.75 and 0.80, which is comparable to the performance of our models (mean accuracy 71%-73.3% and AUC 0.78-0.81). While there is no definite standard for acceptable accuracy, the models in our study recorded accuracy greater than 70% and AUC greater than 0.7, which is better than the accuracy and AUC of past models that used novel predictors, including genetics [20,24], detailed dietary intake [18,21], and objectively measured physical activity [15].

In this study, the overall performance of the ML models, namely XGBoost, RF, and SVM, was found to be similar to logistic regression, as indicated by the overlapping 95% CIs. This corroborates the findings of a systematic review of 71 studies, which concluded that ML did not offer greater performance benefits than logistic regression for clinical prediction models [43]. Specifically, for obesity prediction, Ferdowsy et al [17] employed 8 algorithms, in addition to logistic regression, in a data set that included 21 well-established risk factors for obesity, such as diet, physical activity, lifestyle behaviors, and disease history. Their study recorded the highest accuracy (97%) with the logistic regression model, which outperformed ML algorithms including k-nearest neighbor, RF, a multilayer perceptron, an SVM, a naive Bayes classifier, adaptive boosting, a decision tree, and a gradient boosting classifier for obesity prediction [17]. Kim et al [18] modeled the effects of 7 dietary factors on overweight or obesity status using data from the Korea National Health and Nutrition Examination Survey. That study showed that the predictive accuracy of logistic regression (0.62486) was higher than that of decision trees (0.54026) and similar to that of a deep neural network model of deep learning (0.62496). Taken together, comparative studies that deal with a small number of strong predictor variables [15,17,18,23] suggest that regression models are likely to perform better than, if not as well as, ML models in obesity prediction.

Another possible reason for this similar performance is that the observed relationships among the significant predictors of obesity in this sample may appear linear on the log-odd scale. Hence, logistic regression was not disadvantaged by assuming linearity in these predictors. In this study, we employed 3 nonlinear ML classifiers due to the fact that many variables, including intrapersonal and socioeconomic factors that affect body weight, such as age, sex, and gender, are nonlinear in nature [44]. However, it could be hypothesized that these nonlinear ML algorithms may have been less proficient at modeling the present data set because the data mostly consisted of binary variables (120/165, 73%).

It is important to acknowledge that different ML algorithms may fit and perform differently when used with different data sets. Guided by previous findings that obesity determinants are different for men and women [19,45], we developed separate, sex-stratified models for overweight or obesity status prediction. However, the predictive accuracy of the sex-specific models was similar to the overall or combined models. This suggests that separate prediction models for each sex are not warranted in this Malaysian adult population.

In terms of predictor variables, weight satisfaction appears to be a consistent, novel predictor in all predictive models, together with such well-established risk factors for obesity as ethnicity, age, and gender. Weight satisfaction is an attitudinal component of body image, which reflects individuals’ feelings and thoughts about their weight [46]. The variable “weight_satisfaction_1,” which represents satisfaction or contentment with current body weight, appears to have had the most influential power in the trained model to predict overweight or obesity status (Figure 3).

This novel finding is consistent with previous studies showing that self-perception of body weight is an important determinant of weight management behaviors and lifestyle practices [47]. However, the relationships between weight satisfaction and weight-related behaviors are complex and multifaceted. Depending on sex, race, ethnicity, accuracy of weight perceptions, and psychological factors, weight satisfaction may promote positive diet and physical activity behaviors or lead to maladaptive or unhealthy weight-control or dieting behaviors [48-50]. As weight satisfaction and dissatisfaction appear to be mostly stable in adulthood [51,52], we posit that this subjective variable may be cognitively easier and more reliable to report than body weight and height among adults. This finding supports the usefulness of including weight satisfaction as a proxy for actual weight status in studies and e-surveys, where anthropometry measurements may not be available or feasible.

### Strengths and Limitations

To the best of our knowledge, this is the first study to employ ML to predict overweight or obesity status in an adult working population in Malaysia. This study used rich data from a large annual survey that included a wide, multi-domain set of predictor variables in working adults with a broad range of ages (18 to 88 years) and occupations. Another strength of the study lay in its employment of advanced ML classifiers with careful cross-validation (to avoid model overfitting) and parameter optimization. The variable importance technique afforded novel insights into significant factors that are correlates of overweight or obesity status in a Malaysian working population.

This study was also limited in several ways. First, the study findings do not infer temporality or causality of the observed predictor-obesity relationships due to the use of a cross-sectional design. However, the findings suggest putative variables that could be explored using novel model interpretation techniques such as Shapley additive explanations [53] and could be considered for further testing in longitudinal or trial settings. Second, mislabeling of obesity was likely, due to the reliance on self-reported body weight and height to derive BMI as a surrogate measure of general obesity. Notably, the prevalence of individuals with overweight or obesity in this study (4934/11,803, 41.8%) was lower than the national prevalence of 50.1% [1]. Such errors, or noise, may have reduced the performance of the models. Therefore, the current findings represent conservative estimates of predictive accuracy. Finally, we acknowledge that the generalizability of our models is limited, as validation was based on testing data that came from the same sample. Validating the models with an external data set would more closely approximate the real performance of the prediction models. Future work is needed to confirm the external validity and reproducibility of the models in other data sets, such as the Malaysia’s Healthiest Workplace surveys from 2018 or later.

### Conclusions

Using a multi-domain set of predictors from a large online employee survey, we constructed models that were able to predict overweight or obesity status in a Malaysian working population with moderate to high accuracy. Weight satisfaction was the most prominent factor, followed by ethnicity, age, and gender, in differentiating individuals with overweight or obese status. Among the 3 ML models (XGBoost, RF, and SVM), XGBoost had the highest accuracy and AUC, but the overall performance of all ML-based models was similar to the logistic regression model for obesity prediction.

This study is complementary to and extends the growing literature showing that ML may be used to predict overweight or obesity status based on online survey data with reasonable accuracy. Besides unveiling distinctive factors that influence weight status in this Asian population, this work also produced potential models or algorithms that can be used to screen for overweight or obesity status in community settings, especially when body weight and height data are not available. A natural progression of this study would be to test the performance of the produced models in an external data set to establish the external validity of the findings.

## Appendix

Multimedia Appendix 1 Predictor variables used in the dataset (n=165).

Multimedia Appendix 2 Description of software packages, methods and tuning parameters for model development.

Multimedia Appendix 3 Study sample characteristics (n=16860).

## Abbreviations

AUC: area under the curve
LR: logistic regression
ML: machine learning
RF: random forest
ROC: receiver operating characteristic curve
SVM: support vector machine
XGBoost: extreme gradient boosting

## References

[1] (2020). National Health and Morbidity Survey 2019: Non-communicable diseases, healthcare demand and healthy literacy. Volume I: NCDs – Non-Communicable Diseases: Risk Factors and other Health Problems. Ministry of Health Malaysia.

[2] Lobstein T, Brinsden H, Neveux M (2022). World Obesity Atlas 2022. World Obesity Federation.

[3] Tremmel M, Gerdtham U, Nilsson PM, Saha S (2017). Economic burden of obesity: a systematic literature review. Int J Environ Res Public Health.

[4] Goettler A, Grosse A, Sonntag D (2017). Productivity loss due to overweight and obesity: a systematic review of indirect costs. BMJ Open.

[5] Afshin A, Forouzanfar MH, Reitsma MB, Sur P, Estep K, Lee A, Marczak L, Mokdad AH, Moradi-Lakeh M, Naghavi M, Salama JS, Vos T, Abate KH, Abbafati C, Ahmed MB, Al-Aly Z, Alkerwi A, Al-Raddadi R, Amare AT, Amberbir A, Amegah AK, Amini E, Amrock SM, Anjana RM, Ärnlöv Johan, Asayesh H, Banerjee A, Barac A, Baye E, Bennett DA, Beyene AS, Biadgilign S, Biryukov S, Bjertness E, Boneya DJ, Campos-Nonato I, Carrero JJ, Cecilio P, Cercy K, Ciobanu LG, Cornaby L, Damtew SA, Dandona L, Dandona R, Dharmaratne SD, Duncan BB, Eshrati B, Esteghamati A, Feigin VL, Fernandes JC, Fürst Thomas, Gebrehiwot TT, Gold A, Gona PN, Goto A, Habtewold TD, Hadush KT, Hafezi-Nejad N, Hay SI, Horino M, Islami F, Kamal R, Kasaeian A, Katikireddi SV, Kengne AP, Kesavachandran CN, Khader YS, Khang Y, Khubchandani J, Kim D, Kim YJ, Kinfu Y, Kosen S, Ku T, Defo BK, Kumar GA, Larson HJ, Leinsalu M, Liang X, Lim SS, Liu P, Lopez AD, Lozano R, Majeed A, Malekzadeh R, Malta DC, Mazidi M, McAlinden C, McGarvey ST, Mengistu DT, Mensah GA, Mensink GBM, Mezgebe HB, Mirrakhimov EM, Mueller UO, Noubiap JJ, Obermeyer CM, Ogbo FA, Owolabi MO, Patton GC, Pourmalek F, Qorbani M, Rafay A, Rai RK, Ranabhat CL, Reinig N, Safiri S, Salomon JA, Sanabria JR, Santos IS, Sartorius B, Sawhney M, Schmidhuber J, Schutte AE, Schmidt MI, Sepanlou SG, Shamsizadeh M, Sheikhbahaei S, Shin M, Shiri R, Shiue I, Roba HS, Silva DAS, Silverberg JI, Singh JA, Stranges S, Swaminathan S, Tabarés-Seisdedos Rafael, Tadese F, Tedla BA, Tegegne BS, Terkawi AS, Thakur JS, Tonelli M, Topor-Madry R, Tyrovolas S, Ukwaja KN, Uthman OA, Vaezghasemi M, Vasankari T, Vlassov VV, Vollset SE, Weiderpass E, Werdecker A, Wesana J, Westerman R, Yano Y, Yonemoto N, Yonga G, Zaidi Z, Zenebe ZM, Zipkin B, Murray CJL, GBD 2015 Obesity Collaborators (2017). Health effects of overweight and obesity in 195 countries over 25 years. N Engl J Med.

[6] Bray GA, Kim KK, Wilding JPH, World Obesity Federation (2017). Obesity: a chronic relapsing progressive disease process. A position statement of the World Obesity Federation. Obes Rev.

[7] Swinburn BA, Sacks G, Hall KD, McPherson K, Finegood DT, Moodie ML, Gortmaker SL (2011). The global obesity pandemic: shaped by global drivers and local environments. Lancet.

[8] Ford ND, Patel SA, Narayan KV (2017). Obesity in low- and middle-income countries: burden, drivers, and emerging challenges. Annu Rev Public Health.

[9] DeGregory KW, Kuiper P, DeSilvio T, Pleuss JD, Miller R, Roginski JW, Fisher CB, Harness D, Viswanath S, Heymsfield SB, Dungan I, Thomas DM (2018). A review of machine learning in obesity. Obes Rev.

[10] Birkin M, Wilkins E, Morris MA (2019). Creating a long-term future for big data in obesity research. Int J Obes (Lond).

[11] Chatterjee A, Gerdes MW, Martinez SG (2020). Identification of risk factors associated with obesity and overweight-a machine learning overview. Sensors (Basel).

[12] Scheinker D, Valencia A, Rodriguez F (2019). Identification of factors associated with variation in US county-level obesity prevalence rates using epidemiologic vs machine learning models. JAMA Netw Open.

[13] Colmenarejo G (2020). Machine learning models to predict childhood and adolescent obesity: a review. Nutrients.

[14] Safaei M, Sundararajan EA, Driss M, Boulila W, Shapi'i A (2021). Comput Biol Med.

[15] Cheng X, Lin S, Liu J, Liu S, Zhang J, Nie P, Fuemmeler BF, Wang Y, Xue H (2021). Does physical activity predict obesity-a machine learning and statistical method-based analysis. Int J Environ Res Public Health.

[16] Delnevo G, Mancini G, Roccetti M, Salomoni P, Trombini E, Andrei F (2021). The prediction of body mass index from negative affectivity through machine learning: a confirmatory study. Sensors (Basel).

[17] Ferdowsy F, Rahi KSA, Jabiullah MI, Habib MT (2021). A machine learning approach for obesity risk prediction. CRBS.

[18] Kim H, Lim DH, Kim Y (2021). Classification and prediction on the effects of nutritional intake on overweight/obesity, dyslipidemia, hypertension and type 2 diabetes mellitus using deep learning model: 4-7th Korea National Health and Nutrition Examination Survey. Int J Environ Res Public Health.

[19] Lee BJ, Kim KH, Ku B, Jang J, Kim JY (2013). Prediction of body mass index status from voice signals based on machine learning for automated medical applications. Artif Intell Med.

[20] Lee Y, Christensen JJ, Parnell LD, Smith CE, Shao J, McKeown NM, Ordovás José M, Lai C (2021). Using machine learning to predict obesity based on genome-wide and epigenome-wide gene-gene and gene-diet interactions. Front Genet.

[21] Selya AS, Anshutz D, Giabbanelli P, Mago V, Papageorgiou E (2018). Machine learning for the classification of obesity from dietary and physical activity patterns. Advanced Data Analytics in Health. Smart Innovation, Systems and Technologies, vol 93.

[22] Taghiyev A, Altun A, Caglar S (2020). A hybrid approach based on machine learning to identify the causes of obesity. Journal of Control Engineering and Applied Informatics.

[23] Thamrin SA, Arsyad DS, Kuswanto H, Lawi A, Nasir S (2021). Predicting obesity in adults using machine learning techniques: an analysis of Indonesian basic health research 2018. Front Nutr.

[24] Wang H, Chang S, Lin W, Chen C, Chiang S, Huang K, Chu B, Lu J, Lee T (2018). Machine learning-based method for obesity risk evaluation using single-nucleotide polymorphisms derived from next-generation sequencing. J Comput Biol.

[25] AIA Vitality: The Healthiest Workplace. AIA Group.

[26] Toh B, Akmal A, Syed JS (2017). Malaysia's healthiest workplace: AIA Vitality special report. The EDGE Malaysia.

[27] Malaysian Workforce: Sleepless and Overworked?. AIA Group.

[28] Toh B, Akmal A, Syed Jaafar S (2018). Malaysia's healthiest workplace AIA vitality special report: methodology and demographics. The EDGE Malaysia.

[29] Luo W, Phung D, Tran T, Gupta S, Rana S, Karmakar C, Shilton A, Yearwood J, Dimitrova N, Ho TB, Venkatesh S, Berk M (2016). Guidelines for developing and reporting machine learning predictive models in biomedical research: a multidisciplinary view. J Med Internet Res.

[30] Stevens LM, Mortazavi BJ, Deo RC, Curtis L, Kao DP (2020). Recommendations for reporting machine learning analyses in clinical research. Circ Cardiovasc Qual Outcomes.

[31] (1995). Physical status: the use and interpretation of anthropometry. Report of a WHO Expert Committee. World Health Organization.

[32] Chen KK, Wee S, Pang BWJ, Lau LK, Jabbar KA, Seah WT, Ng TP (2021). Relationship between BMI with percentage body fat and obesity in Singaporean adults - The Yishun Study. BMC Public Health.

[33] Deurenberg-Yap M, Schmidt G, van Staveren WA, Deurenberg P (2000). The paradox of low body mass index and high body fat percentage among Chinese, Malays and Indians in Singapore. Int J Obes Relat Metab Disord.

[34] Cheong KC, Yusoff AF, Ghazali SM, Lim KH, Selvarajah S, Haniff J, Khor GL, Shahar S, Rahman JA, Zainuddin AA, Mustafa AN (2013). Optimal BMI cut-off values for predicting diabetes, hypertension and hypercholesterolaemia in a multi-ethnic population. Public Health Nutr.

[35] Zaher Zaki Morad Mohd, Zambari R, Pheng Chan Siew, Muruga V, Ng B, Appannah G, Onn Lim Teck (2009). Optimal cut-off levels to define obesity: body mass index and waist circumference, and their relationship to cardiovascular disease, dyslipidaemia, hypertension and diabetes in Malaysia. Asia Pac J Clin Nutr.

[36] Aizuddin AN, Chan CM, Anwar AR, Ong YX, Chin K (2021). Performance of body mass index in identifying obesity defined by body fat percentage and hypertension among Malaysian population: a retrospective study. Int J Gen Med.

[37] Kuhn M (2008). Building predictive models in R using the caret package. J Stat Soft.

[38] Chen T, Guestrin C (2016). XGBoost: a scalable tree boosting system. Proceedings of the 22nd ACM SIGKDD International Conference on Knowledge Discovery and Data Mining.

[39] Breiman L (2001). Random forests. Mach Learn.

[40] Vapnik VN (1995). The Nature of Statistical Learning Theory.

[41] Hossin M, Sulaiman MN (2015). A review on evaluation metrics for data classification evaluations. Int J Data Min Knowl Manag Process.

[42] Hosmer JD, Lemeshow S, Sturdivant R (2013). Area under the receiver operating characteristic curve. Applied Logistic Regression, Third Ed.

[43] Christodoulou E, Ma J, Collins GS, Steyerberg EW, Verbakel JY, Van Calster B (2019). A systematic review shows no performance benefit of machine learning over logistic regression for clinical prediction models. J Clin Epidemiol.

[44] Dogbe W, Salazar-Ordóñez Melania, Gil JM (2021). Disentangling the drivers of obesity: an analytical framework based on socioeconomic and intrapersonal factors. Front Nutr.

[45] Hammond R, Athanasiadou R, Curado S, Aphinyanaphongs Y, Abrams C, Messito MJ, Gross R, Katzow M, Jay M, Razavian N, Elbel B (2019). Predicting childhood obesity using electronic health records and publicly available data. PLoS One.

[46] Cash T, Smolak L (2012). Body Image: A Handbook of Science, Practice, and Prevention.

[47] Haynes A, Kersbergen I, Sutin A, Daly M, Robinson E (2018). A systematic review of the relationship between weight status perceptions and weight loss attempts, strategies, behaviours and outcomes. Obes Rev.

[48] Blake CE, Hébert James R, Lee D, Adams SA, Steck SE, Sui X, Kuk JL, Baruth M, Blair SN (2013). Adults with greater weight satisfaction report more positive health behaviors and have better health status regardless of BMI. J Obes.

[49] Millstein RA, Carlson SA, Fulton JE, Galuska DA, Zhang J, Blanck HM, Ainsworth BE (2008). Relationships between body size satisfaction and weight control practices among US adults. Medscape J Med.

[50] Kuk JL, Ardern CI, Church TS, Hebert JR, Sui X, Blair SN (2009). Ideal weight and weight satisfaction: association with health practices. Am J Epidemiol.

[51] Tiggemann M (2004). Body image across the adult life span: stability and change. Body Image.

[52] Quittkat HL, Hartmann AS, Düsing Rainer, Buhlmann U, Vocks S (2019). Body dissatisfaction, importance of appearance, and body appreciation in men and women over the lifespan. Front Psychiatry.

[53] Lundberg SM, Lee SI (2017). A unified approach to interpreting model predictions. NIPS'17: Proceedings of the 31st International Conference on Neural Information Processing Systems.

