# Supplementary material for: Predicting Overweight and Obesity Status Among Malaysian Working Adults With Machine Learning or Logistic Regression: Retrospective Comparison Study
Source: JMIR Form Res. 2022 Dec 7;6(12):e40404. doi: 10.2196/40404 (PMC9773027; doi:10.2196/40404)
Supplement: Multimedia Appendix 1 [file formative_v6i12e40404_app1.docx]

Multimedia Appendix

Predictor variables used in the dataset (n=165).

| **Domain** | **Variable name^a^** | **Description** |
| --- | --- | --- |
| Sociodemographic | hage | Age (in years) |
| (n=44) | region(16) | States of residence |
|  | gender (2) | Sex (male / female) |
|  | carer_1 | Not a primary carer (Yes/No) |
|  | carer_2 | Primary carer of a child (under 18) (Yes/No) |
|  | carer_3 | Primary carer of a disabled child (Yes/No) |
|  | carer_4 | Primary carer of a disable adult (Yes/No) |
|  | carer_5 | Primary carer of an older person (Yes/No) |
|  | carer_6 | Secondary carer (Yes/No) |
|  | carer_7 | Prefer not to say (Yes/No) |
|  | education(8) | Highest education attainment |
|  | marital_status(5) | Marital status |
|  | ethnicity(5) | Ethnicity |
| Job characteristics | main_job(10) | Current occupation |
| (n=54) | income(14) | Individual monthly income before taxes (RM) |
|  | financial_concerns | Rating on financial concerns |
|  | Fin_never | Because of my money situation, I feel like I will never have the things I want in life (Strongly disagree – Strongly agree) |
|  | fin_gettingby | I am just getting by financially (Strongly disagree – Strongly agree) |
|  | fin_concerned | I am concernd that the money I have or will save won’t last (Strongly disagree – Strongly agree) |
|  | fin_left | I have money left over at the end of the month (Strongly disagree – Strongly agree) |
|  | fin_lifecontrol | My finances control my life (Strongly disagree – Strongly agree) |
|  | employment_contract_1 | What kind of employment contract do you have? Permanent (Yes/No) |
|  | employment_contract_2 | What kind of employment contract do you have? Fixed term (Yes/No) |
|  | employment_contract_3 | What kind of employment contract do you have? Temporary (Yes/No) |
|  | employment_contract_4 | What kind of employment contract do you have? Apprenticeship (Yes/No) |
|  | employment_contract_5 | What kind of employment contract do you have? No contract (Yes/No) |
|  | employment_contract_6 | What kind of employment contract do you have? Zero hour contract (Yes/No) |
|  | employment_contract_7 | What kind of employment contract do you have? Self-employed contractor (Yes/No) |
|  | employment_contract_8 | What kind of employment contract do you have? Other (Yes/No) |
|  | irregular_hours | Work irregular hours (Yes/No) |
|  | travel_time | Time spent to travel from home to work (one way) (in minutes) |
|  | home_flexitime | Able to wrok from home (Yes/No) |
|  | location_percQ_1 | Which of the following do you see as your location/s of work? Onsite (Yes/No) |
|  | location_percQ_2 | Which of the following do you see as your location/s of work? Home-based (Yes/No) |
|  | location_percQ_3 | Which of the following do you see as your location/s of work? Remote (Yes/No) |
|  | use_flexitime | Able to work flexible hours (Yes/No) |
|  | desk_work | Proportion of time spent sitting at the desk (5 categories by percent) |
|  | manual_work | Engage in physically demanding labour (Yes/No) |
|  | working_hours | Working hours per week (in hours) |
|  | workhr_week | Actual hours of working last week (in hours) |
|  | sick_leave | Hours missed from work because of health problems (in hours) |
|  | oth_leave | Hours missed from work because of other reasons (in hours) |
|  | prod_quality | In the last four weeks, how often was the quality of your work lower than it should have been? (All of the time - None of the time) |
|  | prod_efficiency | In the last four weeks, how often did you work slower than you should have done? (All of the time - None of the time) |
|  | engaged | Involved in volunteering or other forms of community/civic participation/life (Yes/No) |
| Status perception | job_satisfied | Job satisfaction rating (Strongly agree - Strongly disagree) |
| (n=13) | life_satisfaction | Life satisfaction rating (Not at all – Completely) |
|  | physical_health | Physical health status (Very good – Very poor) |
|  | mental_health | Mental health status (Very good – Very poor) |
|  | weight_satisfaction(3) | Current weight satisfaction (Happy, Not happy with no intention to change, Want to change) |
|  | exercise_satisfaction(3) | Exercise habit satisfaction (Happy, Not happy but don’t want to exercise more, Want to change) |
|  | diet_satisfaction(3) | Diet satisfaction (Happy, Needs improvement but don’t want to change, Want to change diet) |
| Lifestyle-related behaviours | sleep_length | Sleep duration in 24-hour period (in hours) |
| (n=54) | sleep_quality | Sleep quality (Very Good – Very Poor) |
|  | sleep_refreshing | My sleep was refreshing (Not at all - Very much) |
|  | sleep_problem | I had a problem with my sleep (Not at all - Very much) |
|  | sleep_asleep | I had difficulty falling asleep (Not at all - Very much) |
|  | sleep_wake | Frequency of waking up during a typical night’s sleep (times) |
|  | sleep_out | Frequency of getting out of bed during a typical night’s sleep (times) |
|  | sleep_urinate | Frequency of getting up during the night to go to the bathroom (times) |
|  | Sleep_FUSP | Average length of the first period of uninterrupted sleep (hours) |
|  | smoke_status(3) | Smoking status (Never, Previous, Current smoker) |
|  | e_cigs_status(3) | e-cigarette smoking status (Never, Previous, Current smoker) |
|  | alcohol_freq | Frequency of alcohol intake (days per week) |
|  | fruits_serv | Fruit intake (serving per day) |
|  | veg_serv | Vegetable intake (serving per day) |
|  | wholegrain_serv | Whole grain intake (serving per day) |
|  | lean_meats(7) | Choose lean meat over higher fat meats (Never – Always, I don’t eat meat) |
|  | dairy(7) | Choose fat-free over full fat dairy products (Never – Always, I don’t eat dairy products) |
|  | add_fats(7) | Add fats to food after cooking (Never – Always, I don’t eat fat) |
|  | add_salt(6) | Salt added to food per meal (Never, Half teaspoon – A heaped dessert spoon) |
|  | trans_fats | Intake frequency of high trans-fat foods intake (times per day) |
|  | sugary_colddrinks | Intake frequency of cold sugar-sweetened beverages (times per day) |
|  | sugary_hotdrinks | Intake frequency of hot sugar-sweetened beverages (times per day) |
|  | metwalk | Physical activity score of walking (MET-minutes/week)^1^ |
|  | metmod | Physical activity score of moderate-intensity activities (MET-minutes/week) ^1^ |
|  | metvig | Physical activity score of vigorous-intensity activities (MET-minutes/week) ^1^ |
|  | mettotal^b^ | Total physical activity score (metwalk + metmod + mettotal) (MET-minutes/week) |
|  | kesstotal^c^ | Psychological distress score |

^a^Variable name with a number-containing bracket is a categorical variable of each categorical value was subsequently converted into a new binary variable (value 1 or 0) through one-hot encoding.

^b^Based on International Physical Activity Questionnaire [1, 2]

^c^Based on Kessler Psychological Distress Scale, K10 ranging from 0-45 [3]

# References

1. Hallal PC, Victora CG. Reliability and validity of the international physical activity questionnaire (IPAQ). Med Sci Sports Exerc. 2004;36(3):556.

2. IPAQ Research Committee. Guidelines for data processing and analysis of the International Physical Activity Questionnaire (IPAQ)-short and long forms. 2005.

3. Tiong XT, Abdullah NSS, Bujang MA, Ratnasingam S, Joon CK, Wee HL, et al. Validation of the Kessler’s psychological distress scale (K10 & K6) in a Malaysian population. ASEAN Journal of Psychiatry. 2018;19(1).
