# Supplementary material for: Predicting Overweight and Obesity Status Among Malaysian Working Adults With Machine Learning or Logistic Regression: Retrospective Comparison Study
Source: JMIR Form Res. 2022 Dec 7;6(12):e40404. doi: 10.2196/40404 (PMC9773027; doi:10.2196/40404)
Supplement: Multimedia Appendix 2 [file formative_v6i12e40404_app2.docx]

Multimedia Appendix

Description of software packages, methods and tuning parameters for model development.

| **Algorithm** | **Package / Method** | **Parameters** | **Final chosen model** |
| --- | --- | --- | --- |
| Extreme Gradient Boosting | caret / xgbTree | nrounds = c(50, 100, 200, 500)  max_depth = 3:9  eta = 0.1  gamma = 0.1  colsample_bytree = 1  min_child_weight = 1  subsample = 1 | nrounds = 100 max_depth = 3  eta = 0.1  gamma = 0.1 colsample_bytree = 1  min_child_weight = 1 subsample = 1 |
| Random Forest | caret / rf | mtry=1:8 | mtry = 7 |
| Support Vector Machine | caret / svmRadial | sigma = 0.05  C = c(0,0.01, 0.05, 0.1, 0.25, 0.5, 0.75, 1, 1.25, 1.5, 1.75, 2,5) | sigma = 0.003539225  C = 0.25 |
| Logistic Regression | caret / glm |  |  |
