# Supplementary material for: Predicting Overweight and Obesity Status Among Malaysian Working Adults With Machine Learning or Logistic Regression: Retrospective Comparison Study
Source: JMIR Form Res. 2022 Dec 7;6(12):e40404. doi: 10.2196/40404 (PMC9773027; doi:10.2196/40404)
Supplement: Multimedia Appendix 3 [file formative_v6i12e40404_app3.docx]

Multimedia Appendix

Study sample characteristics (n=16860).

| Variables^a^ | **Male (n=6904)** | **Female (n=9956)** | **Total (n=16860)** | **p value** |
| --- | --- | --- | --- | --- |
| Age (in years) |  |  |  | < 0.001^b^ |
| - Mean (SD) | 35.6 (9.8) | 34.2 (9.0) | 34.8 (9.3) |  |
| - Range | 18.0 - 88.0 | 18.0 - 79.0 | 18.0 - 88.0 |  |
| Ethnicity |  |  |  | < 0.001^c^ |
| - Malay | 3266 (47.3%) | 4640 (46.6%) | 7906 (46.9%) |  |
| - Chinese | 2532 (36.7%) | 4078 (41.0%) | 6610 (39.2%) |  |
| - Indian | 700 (10.1%) | 772 (7.8%) | 1472 (8.7%) |  |
| - Bumiputera Sabah/Sarawak | 257 (3.7%) | 328 (3.3%) | 585 (3.5%) |  |
| - Others | 149 (2.2%) | 138 (1.4%) | 287 (1.7%) |  |
| Highest Education Attainment |  |  |  | 0.330^c^ |
| - No formal or primary education | 21 (0.3%) | 21 (0.2%) | 42 (0.2%) |  |
| - Secondary education | 876 (12.7%) | 1235 (12.4%) | 2111 (12.5%) |  |
| - Post secondary non tertiary education | 1394 (20.2%) | 2034 (20.4%) | 3428 (20.3%) |  |
| - University undergraduate (BSc) education | 3570 (51.7%) | 5247 (52.7%) | 8817 (52.3%) |  |
| - University postgraduate (MSc/PhD) education | 1043 (15.1%) | 1419 (14.3%) | 2462 (14.6%) |  |
| Marital status |  |  |  | < 0.001^c^ |
| - Single | 2538 (36.8%) | 4538 (45.6%) | 7076 (42.0%) |  |
| - Married | 4110 (59.5%) | 4844 (48.7%) | 8954 (53.1%) |  |
| - Separated/Divorced | 91 (1.3%) | 276 (2.8%) | 367 (2.2%) |  |
| - Widowed | 28 (0.4%) | 83 (0.8%) | 111 (0.7%) |  |
| - Prefer not to say | 137 (2.0%) | 215 (2.2%) | 352 (2.1%) |  |
| Occupation |  |  |  | < 0.001^c^ |
| - Manager | 1975 (28.6%) | 2381 (23.9%) | 4356 (25.8%) |  |
| - Professional | 2159 (31.3%) | 2420 (24.3%) | 4579 (27.2%) |  |
| - Technician or junior professional | 908 (13.2%) | 759 (7.6%) | 1667 (9.9%) |  |
| - Clerical support worker | 231 (3.3%) | 428 (4.3%) | 659 (3.9%) |  |
| - Service worker | 498 (7.2%) | 2019 (20.3%) | 2517 (14.9%) |  |
| - Sales worker | 259 (3.8%) | 236 (2.4%) | 495 (2.9%) |  |
| - Skilled agricultural forestry and fishery worker | 36 (0.5%) | 2 (0.0%) | 38 (0.2%) |  |
| - Craft and related trades worker | 63 (0.9%) | 16 (0.2%) | 79 (0.5%) |  |
| - Plant and machine operator or assembler | 11 (0.2%) | 8 (0.1%) | 19 (0.1%) |  |
| - Elementary occupations | 764 (11.1%) | 1687 (16.9%) | 2451 (14.5%) |  |
| Individual monthly income |  |  |  | < 0.001^c^ |
| - B40 (Less than RM4,999) | 2297 (33.3%) | 4161 (41.8%) | 6458 (38.3%) |  |
| - M40 (RM5,000 - RM10,999) | 2910 (42.1%) | 4134 (41.5%) | 7044 (41.8%) |  |
| - T20 (RM11,000 and above) | 1019 (14.8%) | 809 (8.1%) | 1828 (10.8%) |  |
| - Prefer not to say | 678 (9.8%) | 852 (8.6%) | 1530 (9.1%) |  |
| BMI status |  |  |  | < 0.001^c^ |
| - Overweight / obese (BMI>=25) | 3430 (48.7%) | 3618 (51.3%) | 7048 (41.8%) |  |

^a^ Data are presented as n (%) or mean (SD).

^b^P value indicate differences between groups calculated using linear model ANOVA test for continuous variables

^c^Pearson’s Chi-squared test. P value indicate differences between groups calculated using Pearson Chi-squared test for categorical variables.
